# Advances in Glaucoma Surgery

**DOI:** 10.3390/jcm12030828

**Published:** 2023-01-20

**Authors:** Karl Mercieca, Michele Figus

**Affiliations:** 1 Glaucoma Unit, University Hospital Eye Clinic, 53127 Bonn, Germany; 2 Faculty of Biology, Medicine and Health, University of Manchester, Manchester M13 9PL, UK; 3 Department of Surgical, Medical, Molecular Pathology and Critical Care Medicine, University of Pisa, 56126 Pisa, Italy

Glaucoma is one of the leading causes of irreversible sight loss worldwide, with a prevalence of 64.3 million in 2013 and an estimated increase to 111.8 million by 2040 [1]. The initial treatment for glaucoma usually comprises topical medication, but further procedures depend on the type of glaucoma, disease stage and severity, and several individual factors, including age, life expectancy, treatment adherence, quality of life, and both patients’ and clinicians’ personal choices. Irrespective of all these factors, surgical intervention may still be required in those patients with significant progression and uncontrolled disease despite maximum medical therapy [2].

Since its inception over 50 years ago, trabeculectomy has been the most common glaucoma surgery performed globally [3], although studies from the United States have demonstrated a decreasing trend in trabeculectomy cases in favor of newer surgical techniques such as minimally invasive or less invasive glaucoma surgical devices [4]. These span a large variety of true microinvasive invasive glaucoma surgeries or MIGS, defined by an ‘ab interno’ intracameral microincisional approach [5,6,7], and bleb-forming sub-conjunctival drainage devices such as the PreserFlo^TM^ Microshunt (Santen, Miami, FL, USA) and Xen-45^TM^ (Abbvie/Allergan, Irvine, CA, USA). The former can further be subdivided into ‘cutting’ MIGSs such as the Kahook Dual Blade^TM^ (KDB, New World Medical, Rancho Cucamonga, CA, USA) and Trabectome^TM^ (NeoMedix Corp., San Juan Capistrano, CA, USA), ‘Trabecular Meshwork stent bypass’ MIGSs such as the iStent^TM^ (Glaukos, Aliso Viejo, CA, USA) and Hydrus^TM^ (Alcon, Fort Worth, TX, USA), and ‘Canaloplasty’ MIGS techniques such as the OMNI^TM^ (Sight Sciences, Menlo Park, CA, USA) and iTrack Advance^TM^ (Nova Eye, Lakes Creek, Queensland, Australia) systems. These techniques have become more popular, particularly in patients who need a reduction in medication burden and also to potentially avoid some of the postoperative complications attributed to conventional glaucoma filtration surgery [8,9]. Additionally, and particularly in Europe, non-penetrating glaucoma surgeries (NPGS), such as deep sclerectomy and canaloplasty, have been developed to provide safer yet still effective ways of reducing intraocular pressure (IOP) in patients with more advanced glaucoma where target eye pressures are significantly lower [10,11,12]. The use of these procedures is increasing significantly compared to conventional trabeculectomy due to the drawbacks of risk of hypotony, intensive post-operative management, the need for proper training, and a significant learning curve. Finally, a further group of glaucoma surgeries, in the form of glaucoma drainage devices (GDDs) or ‘tubes’, have been around for a while as options for complex disease, including secondary glaucoma due to neovascularization and uveitis, congenital and juvenile glaucoma, aphakic glaucoma, etc. [13,14,15]. Although not that new in concept, new GDDs have been developed over the last few years, offering different lumen sizes and plate designs, which can further alter the outcomes we expect from these devices [16,17,18].

Advances in glaucoma surgery have therefore been happening for over the last five decades. However, there has never been such an exciting time to work in the field of glaucoma surgical therapy as the spectrum of options available has become so wide, dense, and varied. Big Pharma is acquiring more and more start-ups that have developed, tried, and tested their products with ever-increasing amounts of evidence building over the last few years. This trend will only increase the drive to seek the best devices and to utilize them to their best capacity in individualized ways. Ultimately, this can only benefit the patient, as long as sound evidence, real-life experience, and cost-effectiveness are also available and considered simultaneously. The evolution of older surgeries such as more modern and innovative ways of performing trabeculectomy and NPGS, and the dissemination of more recent techniques and devices, including their use beyond the original indications for which they were developed, can only boost our chances of effectively lowering IOP and reducing glaucoma visual disability for our patients. We truly hope that this Special Issue, entitled “Advances in Glaucoma Surgery”, can truly capture the essence of these new, exciting, and creative ways of enhancing the effectiveness and safety of our glaucoma surgical armamentarium.
